# The Extracellular Domain of Human High Affinity Copper Transporter (hNdCTR1), Synthesized by *E. coli* Cells, Chelates Silver and Copper Ions In Vivo

**DOI:** 10.3390/biom7040078

**Published:** 2017-11-03

**Authors:** Tatiana P. Sankova, Iurii A. Orlov, Andrey N. Saveliev, Demid A. Kirilenko, Polina S. Babich, Pavel N. Brunkov, Ludmila V. Puchkova

**Affiliations:** 1 Department of Biophysics, Peter the Great St. Petersburg Polytechnic University, Politekhnicheskaya str., 29, St.-Petersburg 195251, Russia; sankova@yandex.ru (T.P.S.); orlov239@gmail.com (I.A.O.); savelev_andrew@mail.ru (A.N.S.); 2 Department of Modern Functional Materials, ITMO University, Kronverksky av., 49, St.-Petersburg 197101, Russia; zumsisai@gmail.com (D.A.K.); brunkov1964@mail.ru (P.N.B.); 3 Center of Nanoheterostructures Physics, Ioffe Institute, Politekhnicheskaya str., 26, St.-Petersburg 194021, Russia; 4 Department of Zoology, Herzen State Pedagogical University of Russia, Kazanskaya str., 6, St.-Petersburg 191186, Russia; babich.polina@gmail.com

**Keywords:** copper transporter 1 metal-binding extracellular domain cloning, copper/silver chelation, *Escherichia coli* filamentous growth, secondary silver nanoparticles formation

## Abstract

There is much interest in effective copper chelators to correct copper dyshomeostasis in neurodegenerative and oncological diseases. In this study, a recombinant fusion protein for expression in *Escherichia coli* cells was constructed from glutathione-S-transferase (GST) and the N-terminal domain (ectodomain) of human high affinity copper transporter CTR1 (hNdCTR1), which has three metal-bound motifs. Several biological properties of the GST-hNdCTR1 fusion protein were assessed. It was demonstrated that in cells, the protein was prone to oligomerization, formed inclusion bodies and displayed no toxicity. Treatment of *E. coli* cells with copper and silver ions reduced cell viability in a dose- and time-dependent manner. Cells expressing GST-hNdCTR1 protein demonstrated resistance to the metal treatments. These cells accumulated silver ions and formed nanoparticles that contained AgCl and metallic silver. In this bacterial population, filamentous bacteria with a length of about 10 µm were often observed. The possibility for the fusion protein carrying extracellular metal binding motifs to integrate into the cell’s copper metabolism and its chelating properties are discussed.

## 1. Introduction

Copper is a structural and catalytic cofactor of vitally essential enzymes [1,2]. Also, it controls activity of ubiquitous and specific transcription factors (e.g., HIF1, p53, Sp1) [3,4,5], participates in cell signaling, cell cycle control, and apoptosis [6,7,8,9,10,11], and it is required for neovascularization [12]. At the same time, copper is a potentially toxic agent, so its ions can initiate Fenton type reactions and produce reactive oxygen species that provoke oxidative stress increasing the risk of development of severe cardiovascular, neurodegenerative, and neoplastic diseases [13,14]. Furthermore, in patients with disorders associated with hepatic copper accumulation, zinc in the DNA-binding domain of the nuclear hormone receptors is replaced by copper; and as a result, the specific binding of hormone receptors to their *cis*-elements is disrupted [15]. In cells and extracellular spaces of multicellular organisms, copper is bound in coordination spheres of cuproenzymes [13], copper transporting [16] or depositing proteins [17]. It can be released when the structure of these proteins is damaged [18]. Therefore, copper chelators are considered as a promising therapeutic agent to scavenge free copper ions in the extra- and intracellular spaces [11,19,20,21,22].

The central member of a safe copper transport into eukaryotic cells is the highly-conserved protein copper transporter 1 (CTR1), which belongs to the family of high affinity Cu(I) transporters [23]. CTR1 is localized in the cell membrane and functions as a homotrimer of 35 kDa glycoprotein subunits. The subunit contains an N-terminal extracellular (ecto)domain (NdCTR1), a transmembrane domain including 3 α-helical motifs, and a short cytosolic C-terminal domain with a copper binding His-Cys-His-motif. The NdCTR1 is *N*- and *O*-glycosylated and contains 66 amino acid residues (in humans), which form a set of three unique metal-binding motifs: His/Met-, His-, and Met-enriched (Swiss-Prot: Q8K211.1). According to the concept of hard and soft acids and bases (Pearson’s hard and soft acid-base (HSAB) principle) they have different presumptive affinity for Cu(II) and Cu(I) [24]. Motifs 1 and 2 are presumably more affine to Cu(II), while motif 3 should be more affine to Cu(I) [25]. CTR1 was shown to bind abiogenic silver ions [26] and cisplatin (a widely used antitumor drug) [27] to transport them into the cells. Ag(I) is isoelectronic to Cu(I) so it is efficiently bound by Cu(I) transporters and carriers; however, it cannot be oxidized in aqueous media, and when included instead of copper to cuproenzymes, it disrupts their structure and catalytic activity [28]. This may be a basis for the use of silver to retard the growth of tumors that require increased amounts of copper [29,30].

The central position of CTR1 as the major pathway for cellular copper uptake in the intestinal epithelium, hepatic and non-hepatic tissue, and particularly tumor cells, as well as its competence to transfer silver and cisplatin, attract considerable attention to the properties of its N-terminal domain. The kinetics of copper, silver and cisplatin binding to metal-binding motifs from NdCTR1 has been studied. Their role as well as the importance of single amino acid residues for copper and cisplatin transport has been evaluated [31,32,33]. However, currently there are no data on NdCTR1 properties as chelator for copper, silver or cisplatin in conditions that model the situation in vivo.

In this work, the ectodomain of human CTR1 (hNdCTR1) was cloned into an expression vector in *Escherichia coli* cells, and the influence of recombinant protein on the sensitivity of *E. coli* cells to silver and copper ions was investigated. The realization that NdCTR1 can chelate copper has prompted the development of copper-specific chelator.

## 2. Materials and Methods

### 2.1. E. coli Strain and Growth Conditions

The study was performed on *E. coli* cells of strain BL21 (DE3) (Stratagene, San Diego, CA, USA) with the genotype *E. coli* B F- dcm ompT hsdS(rB- mB-) gal λ(DE3). The cells were grown aerobically in liquid or agar nutrient medium based on bovine serum hydrolysate (Samson-Med, St. Petersburg, Russia) at 37 °C. Antibiotic (ampicillin, Sigma, Schnelldorf, Germany) was used at the concentration 100 mg/L.

### 2.2. Cloning of N-Terminal Extracellular Domain of hCTR1

The total RNA fraction was extracted from cultured HepG2 cells and used as a template for cDNA synthesis. A 198 bp fragment of the SLC31A1 gene, corresponding to hNdCTR1, was amplified by PCR from the cDNA, using forward and reverse primers (Synthol, Moscow, Russia): 5,cagggatccgatcattcccaccatatggggatg3, and 5,cagctcgagtccagctgtattgatcacca3. The purified fragment (GeneJET PCR Purification Kit, Thermo Scientific, Waltham, MA, USA) was then digested with the restriction enzymes BamH I and Xho I (New England Biolabs, Hitchi, UK) and cloned into glutathione-S-transferase (GST) gene fusion plasmid vector pGEX-4T-1 (Amersham Biosciences, Little Chalfont, UK); the resulting plasmid was named pNdCTR1. *E. coli* strain BL21 (DE3)/pNdCTR1 was obtained by chemical transformation (TransformAid™, Thermo Scientific) of the bacteria. Plasmids were isolated using the alkaline method with GeneJET Plasmid Miniprep Kit (Thermo Scientific). Correctness of the DNA insertion was verified by restriction analysis and direct sequencing. Sequencing was carried out in a MegaBACE 1000 automated sequencer (Molecular Dynamics, Sunnyvale, CA, USA) using a BigDye terminator v1.1 cycle sequencing kit (Applied Biosystems, Warrington, UK). Fusion protein synthesis was induced by isopropyl-β-d-thiogalactoside (IPTG, Sigma, St. Louis, MO, USA). The optimum of IPTG concentration (0.5 mM) and induction time (3.5 h) were determined based on the amount of the target protein in the crude cell extract as determined by PAGE analysis. The protein bands were visualized by Coomassie G250. A commercial mix of proteins with molecular mass from 14 to 200 kDa (Termo Scientific) was used as markers.

### 2.3. Polyacrylamide Gel Electrophoresis, Immunoblotting, and Immunoprecipitation

SDS-PAGE, protein transfer to nitrocellulose membrane (Amersham) and visualization of immunoreactive products were carried out as described earlier [28]. To identify the recombinant protein, antibodies to CTR1 were used. The antibodies were obtained to a chemically synthesized 15-mer peptide corresponding to the second metal-binding domain of hNdCTR1 (P15: 17TMQPSHHHPTTSASH31). The synthesized peptide was conjugated with hemocyanin isolated from red king crab (*Paralithodes camtschaticus*), and the complex was used for immunization of the rabbits. To test the specificity of the obtained antibodies, conjugates of p15 with succinylated bovine serum albumin (BSA) were applied [34]. The antibodies to P15/hemocyanin interacted with modified BSA/P15 and P15 displaced them from the immunocomplex; also, they detected the polypeptide corresponding to mono and trimer of CTR1 on the plasma membrane of murine hepatocytes.

Immunoprecipitation was performed in aliquots of the cell lysate; protein concentration in the samples was equalized by OD280. The antiserum (600 μL) to CTR1 was added to the same volume of the cell lysate and incubated overnight under continuous mixing at 4 °C. Next, 30 μL of goat anti-rabbit IgG (Abcam) were added to the mixture and incubated for 4 h at room temperature. The precipitate was collected by centrifugation and dissolved in pure nitric acid. Silver concentration was measured in both the lysate (100%) and the immunoprecipitate, the quantity (%) of silver bound to hNdCTR1 was calculated.

### 2.4. Treatment of Cells with CuSO_4_ or AgNO_3_

*E. coli* cells were cultured overnight (*E. coli* strain expressing the GST-NdCTR1 fusion protein or the parent GST protein), washed with water and diluted 1:20 in a solution containing CuSO_4_ or AgNO_3_ in various concentrations. Control cell samples were incubated in water. After the treatment, the cells were titrated by 10-fold dilution method to assess cell survival by colony forming units (CFU) on agar plates. Colonies were counted after 24 h incubation at 37 °C and results were expressed using logarithmic notation, where the value shown is the base 10 logarithm of the concentration [35].

### 2.5. Disruption and Fractionation of Bacterial Cells

Cells were washed with water, resuspended and treated with lysozyme (1 mg/mL, Serva, Heidelberg, Germany) for 30 min at room temperature. Next, the cells were disrupted by ultrasound (UZDN-2T, 44 kHz, twelve 30 s cycles with 60 s intervals for cooling) in an ice bath. The soluble cytoplasmic fraction was separated by centrifugation (15,000× *g*, 10 min, 4 °C) and used for chromatographic and immunoprecipitation studies. The samples were equalized by OD280.

### 2.6. Gel-Filtration Chromatography

Bacterial cytosol samples were fractioned by gel-filtration on a Sephacryl S-200 column equilibrated with 20 mM Tris-HCl, 100 mM NaCl buffer, pH 7.6. Detection of the biological material was carried out by spectrophotometry at 280 nm. The void volume of the column was estimated using blue dextran (Sigma). The fractions (~1.5 mL) were collected; GST activity and silver concentration were measured in each fraction. The column was calibrated with blue dextran, electrophoretically pure human ceruloplasmin, 132 kDa (A610/280 = 0.054), isolated by two-stage chromatography [36], BSA, 70 kDa (Sigma), and horse cytochrome C, 13 kDa (Serva).

### 2.7. GST Activity Determination

GST activity was measured with glutathione (Serva) and 1-chloro-2,4-dinitrobenzene (Sigma) according to the Sigma protocol (https://www.sigmaaldrich.com/content/dam/sigma-aldrich/docs/Sigma/Bulletin/cs0410bul.pdf). The change in absorbance at 340 nm was recorded automatically at a room temperature and the GST activity was expressed in terms of change in absorbance per 1 min.

### 2.8. Transmission Electron Microscopy Analysis of Cells

*E. coli* cells were cultured overnight, diluted 25-fold and incubated in aqueous media containing 3.5 μM AgNO_3_ for 1 h at 37 °C. Conventional lacey carbon films suspended on transmission electron microscopy (TEM) copper grids were dampened with the bacterial suspensions, dried in air at room temperature and then studied using Jeol JEM-2100F (accelerating voltage 200 kV, point-to-point resolution 0.19 nm) equipped with an energy-dispersive X-ray spectrometer (EDX) Oxford Instruments INCA. Selected area electron diffraction (SAED) patterns were obtained from areas of about 0.1–1 µm in size using corresponding selective apertures. The characteristic X-ray spectra used for elemental analysis were acquired by focusing the electron beam to a spot ranging from 1 nm to 1 µm in diameter depending on the studied object. Note, that the prominent copper peak in the presented EDX spectra is related to the copper support of TEM specimens.

### 2.9. Measurement of Silver Concentration

Silver concentration was measured by graphite furnace atomic absorption spectrometry (FAAS) with electrothermal atomization and Zeeman correction of nonselective absorption on a ZeeNit P650 spectrometer (Analytik Jena, Jena, Germany) with automatic sampling duplication. Bacterial samples were homogenized in PBS, and dissolved with three volumes of pure concentrated HNO_3_. The silver concentration in chromatographic fractions was measured without additional processing.

## 3. Results

### 3.1. Cloning of hCTR1 Ectodomain

**Figure 1:**
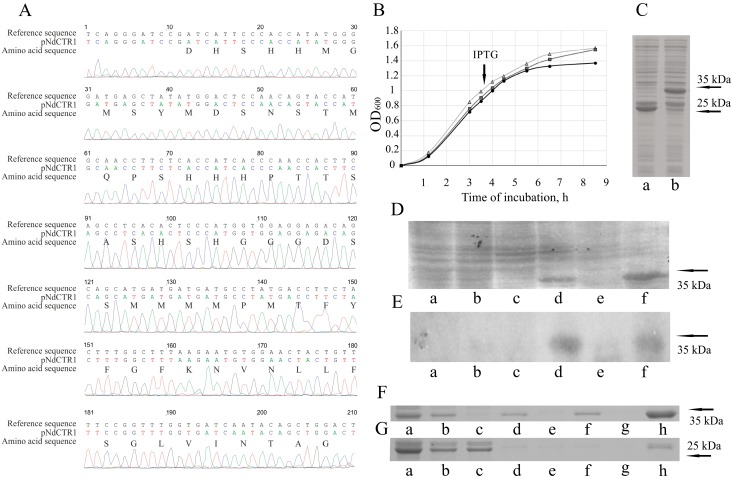
Cloning of ectodomain of human copper transporter 1 (hNdCTR1) and analysis of recombinant fusion protein. (**A**) The correspondence of the cloned nucleotide sequence to the region of the human CTR1 gene. (**B**) The growth rate of the control *Escherichia coli* strain and strains transformed with plasmids expressing either glutathione-S-transferase (GST) or GST-hNdCTR1. Cycles: *E. coli* BL21(DE3); triangles: *E. coli* BL21(DE3)/pGEX-4T-1; squares: *E. coli* BL21(DE3)/pNdCTR1. (**C**) PAG-SDS electrophoresis of crude cellular extracts from *E. coli* BL21(DE3)/pGEX-4T-1 (lane a) and *E. coli* BL21(DE3)/pNdCTR1 (lane b) after incubation with isopropyl-β-d-thiogalactoside (IPTG) for 3.5 h. (**D**) PAG-SDS electrophoresis and (**E**) immunoblotting with antibodies to CTR1 of crude cellular extracts from *E. coli* BL21(DE3)/pNdCTR1 after incubation for 0, 1.5, and 3.5 h. Lanes a, b, c: total protein extract of non-induced culture; lanes d, e, f: total protein extract of IPTG induced culture. (**F**,**G**) PAG-SDS electrophoretic analysis of subcellular fractions from *E. coli* cells transformed with pNdCTR1 and pGEX-4T-1, respectively: cell crude extract (a), cell lysate after ultrasound treatment (b), soluble fraction of lysate obtained by centrifugation for 15 min at 16,000 *g* (c). Insoluble fraction of pellet was 3 times treated with solution containing 1% Triton X-100, 5 mM DTT and 2 M urea. After each treatment, soluble and insoluble fractions were separated by centrifugation for 15 min at 16,000 *g*. Consecutive pellets: d, f, h, and corresponding to them soluble fractions: e, g. arrows show molecular weights.

The DNA fragment, containing 198 bp and corresponding to the full-sized hNdCTR1 was obtained by PCR with reverse transcription of total RNA fraction of HepG2 cells and cloned in pGEX-4T-1 vector as described in the Methods. The correctness of the cloned NdCTR1 inset was verified by direct sequencing (Figure 1A). The growth rates of *E. coli* BL21(DE3) transformed with pGEX-4T-1 or pNdCTR1 cells before and after incubation with IPTG were the same as the growth rate of the non-transformed strain (Figure 1B). IPTG-induced *E. coli* cells bearing the parent pGEX-4T-1 plasmid synthesized a 27.9-kDa polypeptide corresponding to GST (Figure 1C). The BL21 (DE3)/pNdCTR1 *E. coli* cells synthesized a 34.4-kDa polypeptide, and its molecular weight corresponded well to the calculated weight of the fusion protein, which included both GST and 66 amino acid residues of hNdCTR1 (Figure 1C). The maximum production of the fusion protein was observed at 3.5 h after the induction with 0.5 mM IPTG (Figure 1D). The fusion protein contained a region that was recognized by anti-CTR1 antibodies (Figure 1E). Thus, the presented data demonstrate successful expression of the cloned CTR1 ectodomain. After cell disruption, the major part of recombinant protein was found in the fraction known as inclusion bodies, which could not be solubilized under mild conditions of 2 M urea and 1 mM β-mercaptoethanol (Figure 1F). In the cells transformed with pGEX-4T-1 plasmid, GST protein after IPTG induction was found in cytosol (major part) and in pellet (minor fraction). So, fusion protein demonstrated a higher propensity to form the inclusion bodies (Figure 1G).

### 3.2. hNdCTR1 Polypeptide Rescues E. coli Cells from the Toxic Action of Copper and Silver Ions

**Figure 2:**
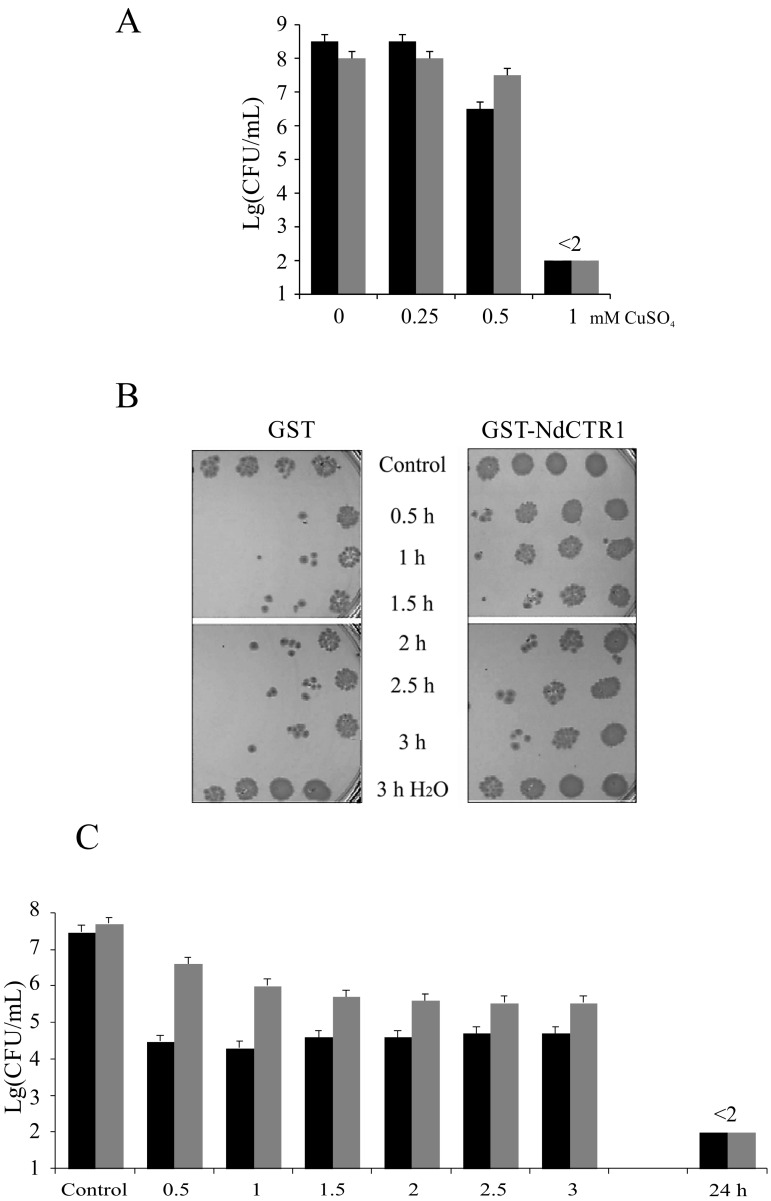
(**A**) Effect of copper ions concentration on *E. coli* BL21(DE3)/pGEX-4T-1 (black bars) and *E. coli* BL21(DE3)/pNdCTR1 (grey bars) after 24 h of treatment. Abscissa: CuSO_4_ concentration, mM; ordinate: CFU, lg. (**B**) Effect of time on survival of *E. coli* BL21(DE3)/pGEX-4T-1 and *E. coli* BL21(DE3)/pNdCTR1 treated with 0.25 mM copper ions (protocol of the experiment). (**C**) Bar graph corresponding to the (**B**) experiment repeated three times. Black bars: *E. coli* BL21(DE3)/pGEX-4T-1; grey bars: *E. coli* BL21(DE3)/pNdCTR1.

Sensitivity of *E. coli* cells to toxic effect of copper and silver ions was evaluated by the conventional method of CFU determination. The experiments were performed in IPTG-induced BL21(DE3)/pGEX-4T-1 or BL21(DE3)/pNdCTR1 strains. The cells were treated with copper sulfate or silver nitrate as described in the Methods. The results indicated that copper ions reduced the number of CFU in the reference strain more efficiently than in the strain producing recombinant hNdCTR1 (Figure 2). The effect depended on copper concentration (Figure 2A) and time of incubation (Figure 2B,C).

**Figure 3:**
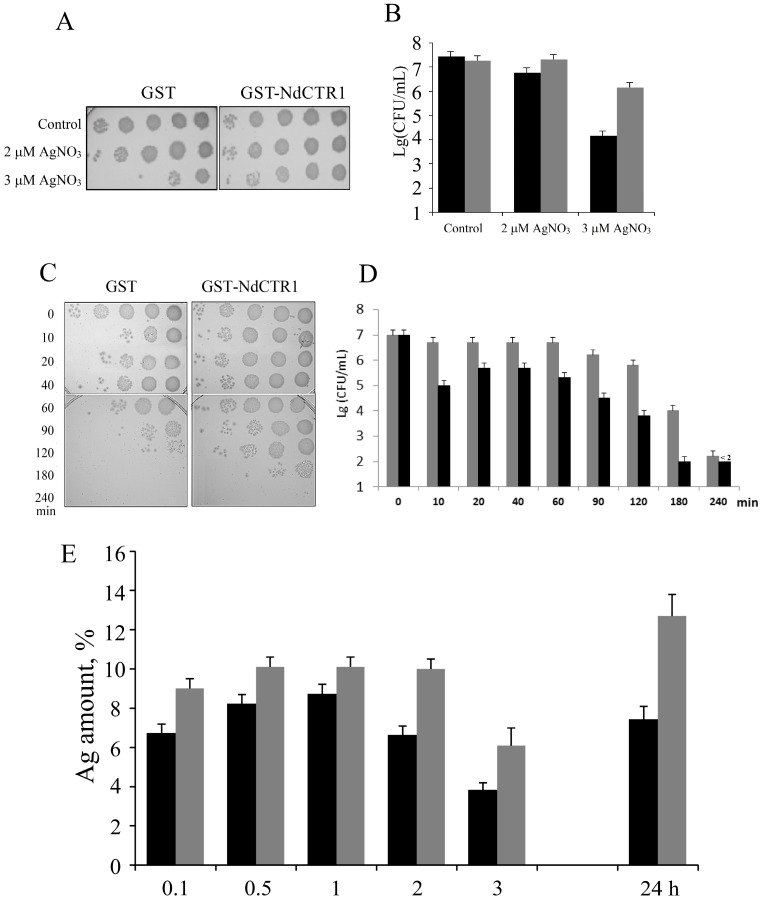
(**A**) Effect of silver ions on *E. coli* Bl21(DE3)/pGEX-4T-1 and BL21(DE3)/pNdCTR1 after 24 h of treatment (protocol of the experiment). (**B**) Bar graph corresponding to the (**A**) experiments repeated three times. Black bars: *E. coli* Bl21(DE3)/pGEX-4T-1; grey bars: BL21(DE3)/pNdCTR1. Abscissa: AgNO_3_ concentration, µM; ordinate: CFU, lg. (**C**) Effect of time on survival of *E. coli* BL21(DE3)/pGEX-4T-1 and *E. coli* BL21(DE3)/pNdCTR1 treated with 3.5 μM AgNO_3_ (protocol of the experiment). (**D**) Bar graph corresponding to the (**C**) experiment treated three times. Black bars: *E. coli* Bl21(DE3)/pGEX-4T-1; grey bars: BL21(DE3)/pNdCTR1. Abscissa: time of treatment, h; ordinate: CFU, lg. (**E**) Dynamic of Ag accumulation by *E. coli* BL21(DE3)/pGEX-4T-1 (black bars) and *E. coli* BL21(DE3)/pNdCTR1 (grey bars) during incubation in 3.5 μM AgNO_3_. Abscissa: time, h; ordinate: CFU, lg.

In the same manner hNdCTR1 rescued *E. coli* cells from toxic effect of silver ions (Figure 3A–D). Two features of the silver ions action on the survival of bacteria attract attention. First, the effect is biphasic: the survival of GST-producing cells dropped in 10 min after the beginning of treatment, then it raised and decreased again at longer periods of treatment. The rescue effect of hNdCTR1 manifested itself at long treatment times (Figure 3D). Second, the rescue effect of hNdCTR1 was associated with silver accumulation. So, the total concentration of silver was two times higher in cells producing hNdCTR1-GST than in the cells expressing parent GST (Figure 3E), and the viability of the former cells was higher (Figure 3B,D).

### 3.3. hNdCTR1 Chelates Silver Ions

**Figure 4:**
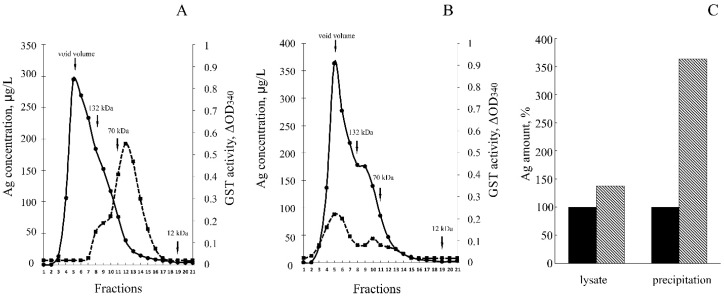
Distribution of silver and GST enzymatic activity in cytoplasm of *E. coli* cells synthetizing GST (**A**) and GST-hNdCTR1 (**B**). Solid line: silver concentration; dotted line: GST enzymatic activity. The details of chromatographic analysis are described in Methods (Section 2.6). The arrows show elution rate of marker proteins: 132 kDa—human ceruloplasmin, 70 kDa—bovine serum albumin, 12 kDa—horse cytochrome *C*. (**С**) Amount of silver precipitated by antibodies to CTR1. Dark bar: *E. coli* BL21(DE3)/pGEX-4T-1; shaded bar: *E. coli* BL21(DE3)/pNdCTR1.

To investigate the ability of hNdCTR1 to bind metal ions we used silver ions as they are considerably easier to track, because they are absent in the cells under normal conditions and transported into the cell and excreted via the same pathways as copper. According to the gel-filtration, in *E. coli* cells expressing the parent pGEX-4T-1 plasmid, GST functional homodimers were observed (Figure 4A).

In the cells synthesizing the fusion protein (Figure 4B), GST activity was also present in fractions, corresponding to GST-hNdCTR1 homodimers (~70 kDa). However, the major part of GST activity was observed in the molecular mass region corresponding to multimers of the fusion protein (more than 110 kDa). The ability of full-sized hCTR1 subunits to form oligomers was described previously, and it was attributed to the properties of the transmembrane domain [37]. The data show that the polypeptide moiety of ectodomain may have its own role in CTR1 oligomerization. It is possible that oligomerization may be a result of the absence of the carbohydrate chains. Also, it cannot be excluded that hydrophobic region located behind the Met-rich motif on the C-terminal of the NdCTR1 (see Figure 1A) is responsible for the oligomerization.

The GST activity of the fusion protein was approximately four times lower than of the parent GST. Most likely, the tendency of the fusion protein to form oligomers partially blocked the access of the substrate to GST active center. Silver atoms were found to be associated with both homodimers and multimers of the fusion protein, but not with GST dimers (Figure 4B). Antibodies to CTR1 precipitated complexes containing silver ions from cellular lysate. The silver content was three–four times higher than in the non-specific precipitate (Figure 4B).

### 3.4. Silver Ions Induced Morphological Changes in E. coli Cells, Which Expressed Fusion Protein

**Figure 5:**
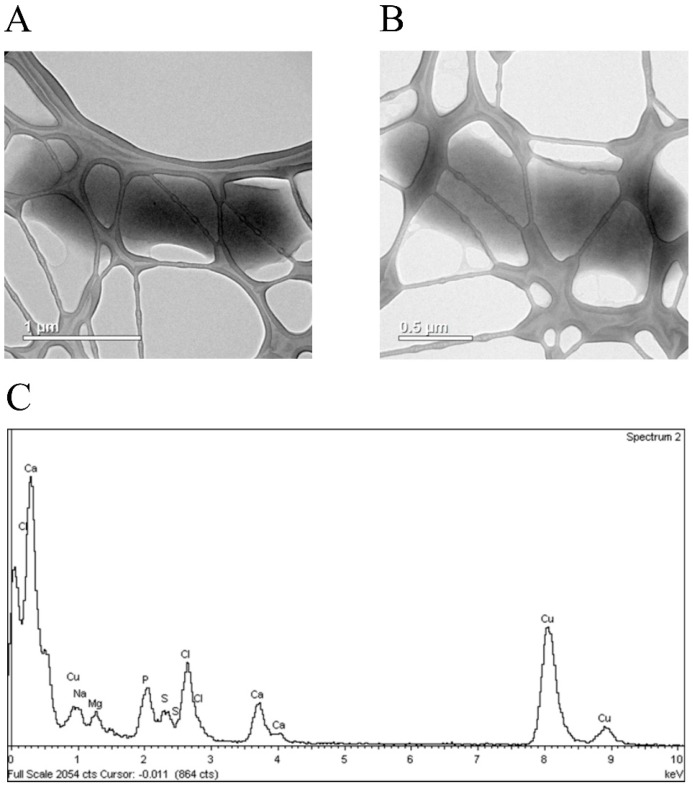
Transmission electron microscopy (TEM) image of *E. coli* cells expressing GST (**A**) or a fusion (GST-hNdCTR1) protein (**B**), which were not treated with silver nitrate. (**C**) Energy-dispersive X-ray spectroscopy (EDS) analysis of cells expressing the fusion protein.

The TEM analysis revealed no morphological changes in the bacterial cells synthesizing GST (Figure 5A) or GST-hNdCTR1 (Figure 5B).

**Figure 6:**
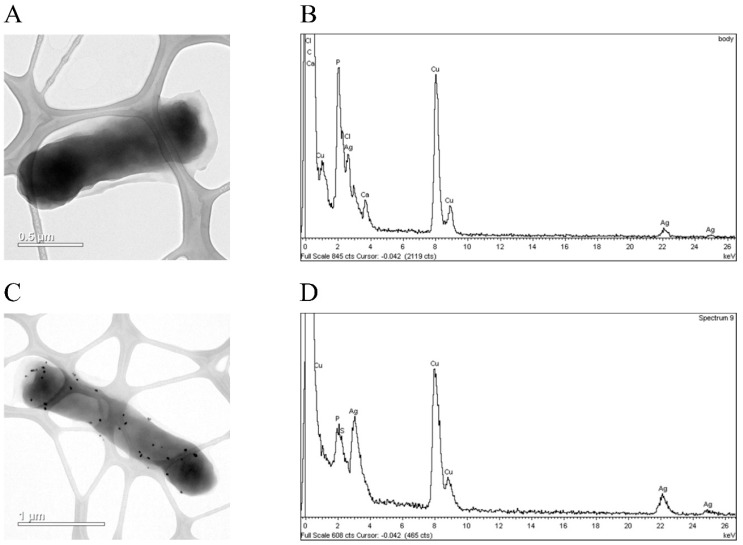
(**A**) TEM images of typical *E. coli* cell expressing GST treated with silver nitrate and (**B**) characteristic EDS spectrum of these bacteria. (**C**) A cell of the same strain, with formed silver nanoparticles (SNP) and (**D**) the characteristic EDS spectrum for cells expressing GST and forming SNP-like corpuscles.

After AgNO_3_-treatment, most of the cells synthesizing GST had the unchanged morphology (Figure 6A), and contained inclusions of silver chloride (Figure 6B). In a single case, silver nanoparticles (SNP) were observed (Figure 6C,D).

**Figure 7:**
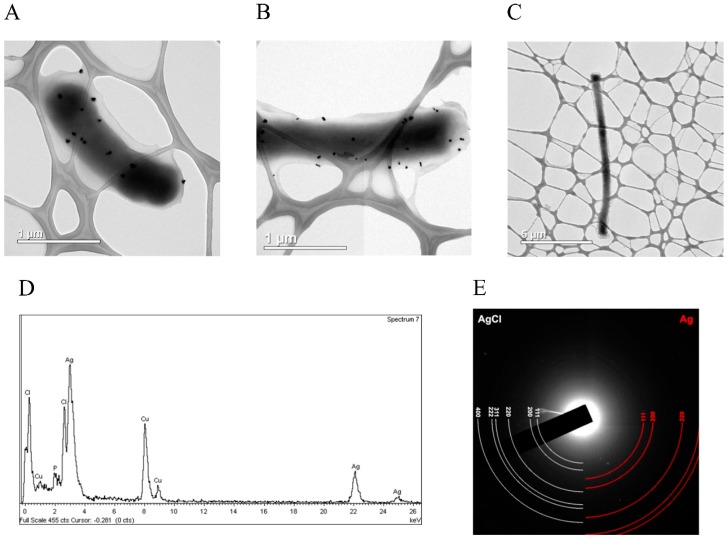
Typical TEM image of silver nitrate-treated *E. coli* cells expressing the fusion (GST-hNdCTR1) protein with AgCl aggregates (**A**) and with formed SNP (**B**). (**C**) Filamentous bacterium. (**D**) Characteristic EDS spectrum of the formed SNP. (**E**) Electron diffraction analysis of the formed SNP.

In the population of the cells synthesizing the fusion protein GST-hNdCTR1, the formation of AgCl aggregates were found in some cases (Figure 7A,D), but the SNP were observed in most of the cells (Figure 7B).

In this cell population, filamentous cells contained SNP with the length exceeding 10 μm were predominant (Figure 7C). Electron diffraction analysis showed that the resulting nanoparticles contained both silver chloride and metallic silver (Figure 7E).

## 4. Discussion

This paper presents data on the specific properties of the extracellular hNdCTR1 inside the bacterial cells. The full-sized hNdCTR1 was cloned by conventional approaches in *E. coli* cells as a part of the GST-fusion protein. This domain’s identity to the human NdCTR1 was confirmed by direct sequencing of the insertion and by immunological assays (Figure 1). The protein was progressively synthesized after induction with IPTG and mostly accumulated in inclusion bodies, although some part of the protein was present in the soluble fraction (Figure 1 and Figure 4). From the inclusion bodies, the fusion protein could be purified on Ni-Sepharose in one step (Figure S1). Expression of the heterogenous protein did not affect the bacterial growth rate (Figure 1B) and cell’s morphology (Figure 5B), however, it saved the bacteria from the toxic effects of copper and silver ions in a dose- and time-dependent manner (Figure 2 and Figure 3). Rescue of bacteria synthesizing protein with hNdCTR1 from silver ions occurred in parallel with silver accumulation (Figure 3F).

In the GST producing cells, after treatment of cells with silver ions, the biphasic pattern of the bacterial viability/time relationship was observed (Figure 3D). *E. coli* has three homeostatic systems that sustain low cytoplasmic copper/silver levels. First, CopA is an ATPase that pumps out copper from the cytoplasm to periplasmic space. Second, CueO is an oxidase that is considered to oxidize periplasmic Cu(I) to Cu(II) thus preventing copper from entering the cytoplasm. CopA and CueO are both induced when the cell is exposed to high copper concentrations. When copper concentrations become critical, the third system, Cus, which pumps out copper from the periplasm to extracellular space, is activated [38]. These systems can be used by bacteria to reduce silver toxicity because many properties of silver and copper ions are similar. In parallel, silver is chelated with high affinity by the recombinant hNdCTR1 and thus detoxified by sequestration from the copper transport routes. The probability of direct hNdCTR1 involvement in bacterial copper transport is very low, as cellular Cu-chaperones contain cysteine-based metal-binding motifs (CXXC) with K_a_ near 10^−18^ for Cu/Ag [26], while NdCTR1 extracellular motifs are completely unrelated to them and do not contain cysteine residues (K_a_ near 10^−6^ for Cu/Ag) [39].

The gel-filtration data (Figure 4A), CTR1 immunoprecipitation studies (Figure 4C) and electron diffraction analysis (Figure 7) are in good agreement with each other and demonstrate that silver is chelated by NdCTR1, resulting in the decrease of its toxic effects (Figure 3). The survival is possibly enhanced by the activation of the stress response, which is manifested by filamentous growth [40] and the appearance of cells longer than 10 μm (Figure 7C). The presented data firmly show that NdCTR1 chelates the excess of copper and silver ions inside the cells. The cell growth is not retarded, so it may be suggested that NdCTR1 does not affect normal cellular copper transport and does not extract copper from the bacterial cuproenzymes.

The ability of hNdCTR1 to chelate metals may be used for practical purposes. Copper dyshomeostasis is a ubiquitous feature of various neurodegenerative, oncology, and cardiovascular diseases, so biogenic chelating agents can be considered as promising candidates for the development of new therapeutic agents [41,42]. NdCTR1 has many favorable properties compared to the chelators used in clinics: (a) it is an ectodomain of a natural human protein that is highly conserved in mammals, it should not display much in-species variability, and thus the induction of an immune response would be unlikely; (b) NdCTR1 is a multifunctional chelator (it can bind Cu(I) and Cu(II), Ag(I), and a series of platinum compounds) [43,44]; (c) it does not contain cysteine residues, meaning that it is not prone to oxidation to disulfides in the extracellular space, where no glutathione reducing system is available; therefore, apo-NdCTR1 can bind copper in the bloodstream and block its transport to tumors; (d) NdCTR1 does not extract copper from cuproenzymes, as copper chelators do [45].

In our work, we observed a conversion of silver ions to SNP (Figure 7). Recently, in vivo study in rats showed that ionic silver after single intravenous injection de novo formed secondary SNP, and the presence of such particles was proven by electron microscopy. However, silver ions were not bioavailable after oral ingestion [46]. At the same time, chronic diet with ionic silver induced formation of Ag0-inclusion in rats [28]. It is possible that Ag^+^ → Ag0 conversion is a common protective mechanism in prokaryotes and eukaryotes against toxic effect of silver ions.

In summary, recombinant hNdCTR1 exhibited a specific chelating ability and was non-toxic. Therefore, it can be considered as a prospectively new therapeutic agent for different disorders that are linked to copper dyshomeostasis.

## Supplementary Materials

The following are available online at www.mdpi.com/2218-273X/7/4/78/s1. Figure S1. GST-NdCTR1 purification on Ni-column. a: initial inclusion bodies fraction; b: molecular weight marker; c-f: void volume; g,h: elution with pH 6.0; i,j: elution with pH 5.0. Inclusion bodies were isolated by several washings of insoluble cellular fraction obtained after cell wall disruption with washing buffer (2M Urea, 0.5% Triton X-100, 150 mM NaCl, 5 mM DTT, 20 mM Tris-HCl pH 7.5). Final pellet was solubilized in 6M Gu-HCl, 50 mM Tris-HCl pH 7.5 solution. After solubilization it was loaded on a preequilibrated with binding buffer (6M Urea, 50 mM Tris-HCl, 0.5 M NaCl, 50 mM NaAc pH 8.0) Ni-IDA-Sepharose IMAC column. Elution was performed with pH step-gradient (6.0, 5.0, 4.0). Elution buffer: 6 M Urea, 50 mM Tris-HCl, 0.5 M NaCl, 50 mM NaAc, pH 8.0.
