# Supplementary material for: The Extracellular Domain of Human High Affinity Copper Transporter (hNdCTR1), Synthesized by E. coli Cells, Chelates Silver and Copper Ions In Vivo
Source: Biomolecules. 2017 Nov 3;7(4):78. doi: 10.3390/biom7040078 (PMC5745460; doi:10.3390/biom7040078)
Supplement: Supplementary file 1 [file biomolecules-07-00078-s001.pdf]

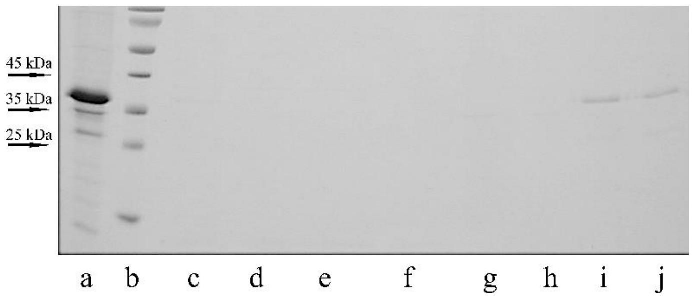

Figure S1. GST-NdCTR1 purification on Ni-column. a: initial inclusion bodies fraction; b: molecular weight marker; c-f: void volume; g,h: elution with pH 6.0; i,j: elution with pH 5.0. Inclusion bodies were isolated by several washings of insoluble cellular fraction obtained after cell wall disruption with washing buffer (2M Urea, 0.5% Triton X-100, 150 mM NaCl, 5 mM DTT, 20 mM Tris-HCl pH 7.5). Final pellet was solubilized in 6M Gu-HCl, 50 mM Tris-HCl pH 7.5 solution. After solubilization it was loaded on a preequilibrated with binding buffer (6M Urea, 50 mM Tris-HCl, 0.5 M NaCl, 50 mM NaAc pH 8.0) Ni-IDA-Sepharose IMAC column. Elution was performed with pH step-gradient (6.0, 5.0, 4.0). Elution buffer: 6M Urea, 50 mM Tris-HCl, 0.5 M NaCl, 50 mM NaAc, pH 8.0.
